# Supplementary material for: Content-rich biological network constructed by mining PubMed abstracts
Source: BMC Bioinformatics. 2004 Oct 8;5:147. doi: 10.1186/1471-2105-5-147 (PMC528731; doi:10.1186/1471-2105-5-147)
Supplement: Additional File 2 — The original results of the above study (non-essential files are deleted to keep the file size under the limit set by BMC bioinformatics). [file 1471-2105-5-147-S2.bz2 › chilibotAdditionalFile2/dip50/663ID9450761E11056/html/NFKB1_RELA.html]

 


 **NFKB1** and **RELA** 
  
Found 53 abstracts in PubMed, retrieved 50.  
 

 What does Google say? 
 PDF only 
| .edu only 

---

**Interactive relationship** (e.g. stimulation, inhibition, etc)

**Neutral relationship**- In addition, NF kappa B p65  [ **RELA** ]   **RelA** , but not NF kappa B p50  **NFKB1** , binds specifically to the NF kappa B site.  Ref: 8207232 J Immunol, 1994
- five independent genes,  **NFKB1**  p105, NFKB2 p100,  **RelA**  p65  [ **RELA** ] , c rel and relB, that encode related proteins that bind to kappa B DNA elements have been isolated.  Ref: 8441377 Mol Cell Biol, 1993
- Immunosupershift and UV cross linking established that the keratinocyte nuclear binding protein of site B 5 GGGGCTTTCC 62 to 253 bp was NFkB  [ **NFKB1** ]  consisting of the p65  [ **RELA** ]  and p50 subunits.  Ref: 7525574 J Biol Chem, 1994
- Our previous work demonstrates that the Rel NF kappa B family proteins c Rel,  **RelA**  p65  [ **RELA** ] , and  **NFKB1**  p50 are involved in the complex that binds to the CD28RE.  Ref: 7623820 Mol Cell Biol, 1995
- Further characterization has revealed the activation and interaction of C EBP alpha, C EBP beta NF IL6,  **NFKB1**  p50, and  **RelA**  p65  [ **RELA** ]  to their specific binding elements present in the bovine IL 6 gene.  Ref: 7669256 DNA Cell Biol, 1995
- In comparison to the conventional GC box containing Sp1 motif, the alpha V kappa B motif also binds in vitro to c Rel and  **RelA**  but not to  **NFKB1** .  Ref: 8572570 Anticancer Res< MedlineTA>Anticancer Res, 1995
- In extracts from activated murine macrophages and macrophage cell lines this element binds several non canonical NF kappa B Rel complexes, in addition to p50  **NFKB1**  homodimer and p50 p65  [ **RELA** ]  NKFB1  **RelA**  heterodimer.  Ref: 7624137 Oncogene, 1995
- A fusion protein that contains 34 N terminal amino acids of  **NFKB1**  and 264 amino acids of  **RelA**  displayed preferential binding to an  **NFKB1**  selective DNA motif while dimerizing with the characteristics of  **RelA** .  Ref: 8321192 Mol Cell Biol, 1993
- Nuclear protein binding to the NFkB  [ **NFKB1** ]  binding sequence was altered by antibodies to the p50 and p65  [ **RELA** ]  components of NFkB  [ **NFKB1** ] .  Ref: 8554599 Biochem Biophys Res Commun, 1995
- Furthermore, substitution of four amino acids from  **NFKB1**  into  **RelA**  was able to alter the DNA binding specificity of the  **RelA**  protein to favor interaction with the  **NFKB1**  selective site.  Ref: 8321192 Mol Cell Biol, 1993
- One nucleoprotein complex contained prototypical NFKB  [ **NFKB1** ]  comprising of p50 and p65  [ **RELA** ]  subunits and a second contained the p65  [ **RELA** ]  subunit and an 80 K factor, p80.  Ref: 8074713 Biochem Biophys Res Commun, 1994
- In supershift assays using NF kappa B specific antibodies, complex C2 was identified to be a p50 homodimer KBF1  [ **NFKB1** ]  and complex C1 was identified as a p50 p65  [ **RELA** ]  heterodimer.  Ref: 8005663 Infect Immun, 1994
- This kappa B motif binds NF kappa B p50  **relA** , p50 c rel and  **relA**  c rel heterodimers as well as KBF1  [ **NFKB1** ]  p50 homodimer.  Ref: 8262046 EMBO J, 1993
- We show that selective inhibition of  **RelA**  NF kappaB decreased induction of  **NFKB1** , interleukin 2, and interleukin 2Ralpha genes but not c myc.  Ref: 9417120 J Biol Chem, 1998
- While authentic p50 homodimers previously termed KBF1  [ **NFKB1** ]  have been detected in nuclear extracts from nontransfected cells, experimental evidence supporting the existence of p65  [ **RELA** ]  homodimers in vivo was lacking.  Ref: 8246997 Mol Cell Biol, 1993
- Virus infection does not appear to alter the amount of  **RelA**  p65  [ **RELA** ]  or  **NFKB1**  p50 but rather affects the capacity of I kappa B alpha to sequester  **RelA**  p65  [ **RELA** ] , therefore leading to constitutive levels of  **RelA**  DNA binding activity and to increased levels of NF kappa B dependent gene activity.  Ref: 8394446 J Virol, 1993
- Recombinant I kappa B alpha MAD 3 inhibited the DNA binding activity of p65  [ **RELA** ] , p49 p65  [ **RELA** ] , and p50 p65  [ **RELA** ]  but stimulated the binding of NFKB2 p49 or  **NFKB1**  p50.  Ref: 8441377 Mol Cell Biol, 1993

**Non-interactive relationship** (e.g. studied together, co-existance, homology, etc.)

- MS association was excluded for the  **NFKB1**  and NFKB3  [ **RELA** ]  genes, which show remarkably low degrees of polymorphism.  Ref: 12058256 Genes Immun, 2002
- Gel mobility shift analysis with in vitro translated and purified proteins indicated that whereas the kappa B element in the human immunodeficiency virus type 1 long terminal repeat bound to all members of the kappa B Rel family examined, the IL 8 kappa B site bound only to  **RelA**  and to c Rel and NFKB2 homodimers, but not to  **NFKB1**  homodimers or heterodimers of  **NFKB1**   **RelA** .  Ref: 8413215 Mol Cell Biol, 1993
- NFKB  [ **NFKB1** ]  2 p52 differs from NFKB  [ **NFKB1** ]  1p50 in its differential affinity for kappa B sequences by itself it binds H2 HLA kappa B sites more efficiently than HIV IgK kappa B sites, while it can bind both sites efficiently when complexed with Rel A p65  [ **RELA** ] .  Ref: 8108136 Oncogene, 1994
- Only antibody to  **RelA**  p65  [ **RELA** ] , but not to  **NFKB1**  p50, NFKB2 p50B, c Rel, or RelB was able to abolish binding, suggesting that  **RelA**  is a major component in these kappa B binding complexes.  Ref: 8413215 Mol Cell Biol, 1993
- We showed that CD28 costimulation accelerated the kinetics of nuclear translocation of c Rel and its phosphorylated form, p50  **NFKB1** , and p65  [ **RELA** ]   **RelA** .  Ref: 7969133 Mol Cell Biol, 1994
- Antisense oligonucleotides to  **RelA** , but not  **NFKB1** , inhibited phorbol myristate acetate induced IL 8 production in Jurkat T lymphocytes.  Ref: 8413215 Mol Cell Biol, 1993
- Transient cotransfection experiments demonstrated that  **RelA**  and  **NFKB1**  expression maximally stimulated HIV 1 LTR and NF kappa B dependent reporter genes.  Ref: 8394446 J Virol, 1993
- A combination of  **RelA**  p65  [ **RELA** ]  and  **NFKB1**  p50 also activated the IL 2 promoter through the CD28RE site.  Ref: 7623820 Mol Cell Biol, 1995
- Purification of this DNA binding complex demonstrated that NF kappaB is a heterodimer composed of two subunits,  **NFKB1**  and  **RelA** .  Ref: 9116500 Protein Expr Purif, 1997
- Binding sites for the NF kappa B transcription factor complex, composed of two subunits, p50  **NFKB1**  and p65  [ **RELA** ]  rel A, are present in many cell adhesion molecules, cytokines, and growth factor receptors.  Ref: 7727726 In Vivo< MedlineTA>In Vivo, 1997
- Over a 24 hr period of cytokine stimulation the p50 p65  [ **RELA** ]  complex of NFKB  [ **NFKB1** ]  was present in the nucleus whilst the second complex declined in abundance after two hours due to the decline of p80.  Ref: 8074713 Biochem Biophys Res Commun, 1994
- The most well characterized form of NF kappa B is comprised of a heterodimer of a 50 kD p50  **NFKB1**  and a 65 kD p65  [ **RELA** ]   **RelA**  protein.  Ref: 8152812 Oncogene, 1994
- Antisense inhibition of the  **RelA**  subunit of NF kappa B transcription factor but not the  **NFKB1**  subunit causes pronounced inhibition of tumor cell growth in vitro and in vivo.  Ref: 8615671 Anticancer Res< MedlineTA>Anticancer Res, 1994
- LY294002, PI3K DN, or AKT DN inhibited NFkB  [ **NFKB1** ]  activation, p65  [ **RELA** ]  transactivation, and cyclooxygenase 2 COX 2 expression induced by lauric acid or constitutively active CA TLR4.  Ref: 12865424 J Biol ChemJ Biol Chem, 1994
- Cotransfection with various NF kappa B subunits indicated that  **RelA**  and c Rel, but neither  **NFKB1**  nor heterodimeric  **NFKB1**   **RelA** , was able to activate transcription from the IL 8 promoter.  Ref: 8413215 Mol Cell Biol, 1993
- Antisense phosphorothioate oligomers to  **relA**  but not  **NFKB1**  caused a rapid inhibition of cell adhesion in diverse cell types.  Ref: 8687102 Anticancer Res< MedlineTA>Anticancer Res, 1993
- To investigate whether transcription factors of the NF kappa B family could play a role in early mammalian development, we have analyzed the expression of  **nfkb1** , nfkb2, c Rel,  **RelA** , RelB, and bcl 3 from 6.5 to 10.5 day mouse embryo implantation sites.  Ref: 10402669 Dev Genet, 1999
- The HLA A enhancer was found to contain two Rel KBF  [ **NFKB1** ]  NF kappa B binding motifs, while the HLA B enhancer has only one and is transactivated less well by overexpression of the NF kappa B p65  [ **RELA** ]  subunit.  Ref: 8265591 Proc Natl Acad Sci U S A, 1993
- Although apigenin did not inhibit TNF alpha induced nuclear translocation of NF kappa B p50  **NFKB1**  p65  [ **RELA** ]   **RelA**  we found this flavonoid did inhibit TNF alpha induced beta galactosidase activity in SW480 cells stably transfected with a beta galactosidase reporter construct driven by four NF kappa B elements, suggesting an action on NF kappa B transcriptional activation.  Ref: 7543732 Am J Pathol, 1995
- also, combinations of  **NFKB1**  p50 and  **RelA**  p65  [ **RELA** ]  or c Rel and  **RelA**  were strong transcriptional activators of reporter gene activity.  Ref: 8413223 Mol Cell Biol, 1993
- Analysis of NF kappa B mRNA in human umbilical vein endothelial cells revealed that  **nfkb1** , nfkb2, and  **relA**  NF kappa B but not c rel were induced by tumor necrosis factor alpha and lipopolysaccharide, which also induce VCAM 1.  Ref: 7692229 Mol Cell Biol, 1993
- Acquisition of  **NFKB1**  selective DNA binding by substitution of four amino acid residues from  **NFKB1**  into  **RelA** .  Ref: 8321192 Mol Cell Biol, 1993
- Antisense inhibition of the  **RelA**  subunit but not the  **NFKB1**  subunit of NK kappa B transcription factor results in a block of cellular adhesion and inhibition of tumor cell growth in vitro and in vivo.  Ref: 8572570 Anticancer Res< MedlineTA>Anticancer Res, 1993
- Probes representing the 300 region or the NF kappa B site alone interacted with NF kappa B proteins present in phorbol myristate acetate, lipopolysaccharide, or Sendai virus induced myeloid cell extracts as well as recombinant  **NFKB1**  p50 and  **RelA**  p65  [ **RELA** ] .  Ref: 8413223 Mol Cell Biol, 1993
- The active complex is composed of two subunits designated  **NFKB1**  and  **RelA**  formerly called p50 and p65  [ **RELA** ] , respectively.  Ref: 8687102 Anticancer Res< MedlineTA>Anticancer Res, 1993
- Furthermore, cotransfection of  **NFKB1**  and  **RelA** , although able to support activation from the human immunodeficiency virus type 1 long terminal repeat, failed to activate expression from the IL 8 promoter.  Ref: 8413215 Mol Cell Biol, 1993
- We now show that a series of topoisomerase poisons actinomycin D, camptothecin, daunomycin and etoposide also activate NF kappaB  **NFKB1**   **RelA**  dimer in ACH 2 and CEM cells.  Ref: 8932379 Nucleic Acids Res, 1996
- We demonstrated a long term induction of p50 p65  [ **RELA** ]  heterodimer, a putative p65  [ **RELA** ]  c Rel heterodimer, and a constitutive nuclear expression of KBF1  [ **NFKB1** ]  p50 homodimers.  Ref: 7905283 Cell Growth Differ, 1993
- The subunits of NF kappa B,  **NFKB1**  formerly p50 and  **RelA**  formerly p65  [ **RELA** ] , belong to a growing family of transcription factors that share extensive similarity to the c rel proto oncogene product.  Ref: 8321192 Mol Cell Biol, 1993
- By using  **NFKB1**  and  **RelA**  subunit fusion proteins, different regions within the RHD were found to confer DNA binding and multimerization functions.  Ref: 8321192 Mol Cell Biol, 1993
- Co expression of p50 suppressed the transactivation by p65  [ **RELA** ]  presumably by competitive DNA binding of transcriptionally inactive p50 dimers KBF1  [ **NFKB1** ] .  Ref: 1935902 EMBO J, 1991
- The virally induced 90 to 100 kDa proteins have a distinct binding specificity for the PRDII domain and an AT rich sequence but do not cross react with NF kappa B subunit specific antisera directed against  **NFKB1**  p105 or p50, NFKB2 p100 or p52,  **RelA**  p65  [ **RELA** ] , or c rel.  Ref: 8394446 J Virol, 1993
- The gel supershift assay with  **Nfkb1** ,  **Rela**  and or Rel antibodies revealed that the specific molecular forms of NF kappaB activated by radiation in the spleen were  **Nfkb1**  homodimers and  **Nfkb1**   **Rela**  heterodimers.  Ref: 10360790 Radiat Res, 1999
- We demonstrate that  **RelA** , either as a homodimer or a heterodimer with some other members of the Rel family and not the classical NF kappa B  **RelA**   **NFKB1** , is involved in the differential growth control of tumor cells.  Ref: 8615671 Anticancer Res< MedlineTA>Anticancer Res, 1999
- Using antibodies, we demonstrated that these complexes contain NFkappaB subunits  **NFkB1** , NFkB2,  **RelA**  and c Rel.  Ref: 10773315 Virus Res, 2000
- In this study, we have analyzed the roles of the nuclear factors KBF1  [ **NFKB1** ]  p50 and p65  [ **RELA** ]  in regulation of class I expression in high and low metastatic tumor cells.  Ref: 8496683 J Exp Med, 1993
- Within the  **NFKB1**  portion of this fusion protein, a single amino acid change of His to Arg altered the DNA binding specificity to favor interaction with the  **RelA**  selective DNA motif.  Ref: 8321192 Mol Cell Biol, 1993
- Low class I expressing cells show at higher levels of KBF1  [ **NFKB1** ]  p50 and NF kappa B p50 p65  [ **RELA** ]  binding activity than high class I expressing cells.  Ref: 8496683 J Exp Med, 1993
- In bone marrow, an NF kappaB like binding factor was induced that may be  **Nfkb1**   **Rela**  and Rel  **Rela**  like heterodimers, but it exhibited a higher mobility than  **Nfkb1**  homodimers.  Ref: 10360790 Radiat Res, 1999
